# Effects of insularity on genetic diversity within and among natural populations

**DOI:** 10.1002/ece3.8887

**Published:** 2022-05-07

**Authors:** David A. G. A. Hunt, Joseph D. DiBattista, Andrew P. Hendry

**Affiliations:** ^1^ 5620 Redpath Museum and Department of Biology McGill University Montreal Quebec Canada; ^2^ Australian Museum Research Institute Australian Museum Sydney New South Wales Australia

**Keywords:** conservation, genetic diversity, insularity, isolation, population size

## Abstract

We conducted a quantitative literature review of genetic diversity (GD) within and among populations in relation to categorical population size and isolation (together referred to as “insularity”). Using populations from within the same studies, we were able to control for between‐study variation in methodology, as well as demographic and life histories of focal species. Contrary to typical expectations, insularity had relatively minor effects on GD within and among populations, which points to the more important role of other factors in shaping evolutionary processes. Such effects of insularity were sometimes seen—particularly in study systems where GD was already high overall. That is, insularity influenced GD in a study system when GD was high even in non‐insular populations of the same study system—suggesting an important role for the “scope” of influences on GD. These conclusions were more robust for within population GD versus among population GD, although several biases might underlie this difference. Overall, our findings indicate that population‐level genetic assumptions need to be tested rather than assumed in nature, particularly for topics underlying current conservation management practices.

## Graphical abstract

We conducted a quantitative literature review of genetic diversity (GD) within and among populations in relation to categorical population size and isolation (together referred to as “insularity”). Contradictory to typical expectations, we found that insularity had relatively minor effects on GD within and among populations, which points to the more important roles of other factors in shaping evolutionary processes. Our findings indicate that population‐level genetic assumptions need to be tested rather than assumed in nature, particularly for topics underlying current conservation management practices.

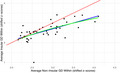

## 1 INTRODUCTION

An insular environment or “island” is any area of habitat suitable for a specific ecosystem that is surrounded by an expanse of unsuitable habitat. Examples of insular systems include mountain tops, lakes, seamounts, enclosed seas, and isolated islands or reefs. These systems have several important properties that set them apart from non‐insular systems and thus dictate their specific consideration in this assessment. … Many of these problems facing insular taxa are compounded when the insular habitats are very small and isolated, including tiny remote Pacific islands, alpine lakes, and desert oases. … Finally, the small population sizes typical of species living in small insular habitats can lead to genetic drift and inbreeding that greatly reduce genetic variation in some situations. As insular taxa are often very local, rare, unique, and vulnerable, active and specific conservation efforts are critical. (IPBES, 2019, p. 218)

The UN Global Assessment of Biodiversity and Ecosystem Services (IPBES, 2019) emphasized the importance of formal consideration of systems considered to be “insular” owing to their inherent vulnerability. As the above quote exemplifies, such insular populations—especially when they are small—are expected to suffer from lower levels of genetic diversity (GD). The last author of the present paper (A. P. Hendry) helped prepare the global assessment and—in so doing—was encouraged to undertake a quantitative assessment of the genetic properties of small and isolated populations considered by authorities on those systems to fall into this “insular” category. In the present paper, we report on the results of that quantitative assessment, starting with a review of the generally expected effects of isolation and small habitats on genetic variation within and among populations.

Genetic diversity within a species has been highlighted as a level of biodiversity worth protecting (Des Roches et al., 2018; Leigh et al., 2019; Millet et al., 2019; Mimura et al., 2017). GD is the raw material that fuels organisms' evolutionary responses to changing environments, such as those imposed by climate change, pollution, or invasive species. GD is thus a key to “evolutionary rescue” wherein rapid adaptation to environmental change reverses the initial fitness declines that accompany severe or rapid environmental change (Carlson et al., 2014; Gomulkiewicz & Holt, 1995; Hendry et al., 2018). GD is also key to “genetic rescue,” wherein alleles introduced by migration reduce inbreeding depression in small and isolated populations (Whiteley et al., 2015). GD also can have important direct effects on entire populations and communities, thus shaping ecosystem services, sustainability, and nature's contributions to people (Díaz et al., 2018; Faith et al., 2010; Hendry, 2017; Naeem et al., 2016; Rudman et al., 2017; Stange et al., 2021). In recognition of these important roles of GD, biologists have championed the idea of identifying populations fit for conservation based on their GD (Coates et al., 2018; Petit et al., 1998), and then preserving and enhancing GD as a downstream conservation method (Paz‐Vinas et al., 2018). However, this view is not without challenge (Teixeira & Huber, 2021), and such endeavors are critically dependent on understanding the factors shaping GD within and among populations.

In idealized theoretical models, the speed at which alleles are eliminated from a population is inversely related to population size through the effects of genetic drift (Charlesworth, 2009). In contrast, the rate that new alleles are added to a population is positively related to population size due to mutational inputs (Kirby, 1975). As a result, relatively smaller and more isolated populations (herein “insular”) are expected to support lower GD *within* populations, for example, having fewer alleles and lower heterozygosity (Frankham, 1996). These same conditions are expected to generate greater GD *among* populations (e.g., greater allele frequency differences)—owing to independent genetic drift and selection within those populations. Insular systems contain a large proportion of endemic species (Wilmé et al., 2006), largely because the colonists are rare, leaving “empty niches” into which the colonizing species can exploit and radiate. Insular populations often have a narrow range of environmental conditions to which local organisms are precisely adapted. As a result, changing environmental conditions (e.g., climate warming) can eliminate suitable habitats without the option of movement or adaptive responses (Corlett & Westcott, 2013; Courchamp et al., 2014).

Specific empirical studies certainly support the above‐mentioned expectations of lower GD within insular populations (Crispo et al., 2006; Soro et al., 2017; Stow et al., 2006), and yet other studies yield contradictory outcomes. For instance, Kuo and Janzen (2004) describe an isolated population where “[a] bottleneck had little effect on its level of genetic diversity,” which was hypothesized to be the result of specific life histories (late age‐at‐maturity and long lifespans) in ornate box turtles (*Terrapene ornata*) resulting in relatively slow changes to GD. Similarly, Hailer et al. (2006) described a situation where “[...] long generation time [...] has acted as an intrinsic buffer against loss of genetic diversity, leading to a shorter effective time of the experienced bottleneck.” In another example, only modest declines in GD were seen in some pinniped species under intensive harvesting (Stoffel et al., 2018), although the same study saw large declines in GD in other pinniped species. Additional studies found “no significant relationship between population size and levels of heterozygosity” (Bezemer et al., 2019). The fact that the distribution of expected GD is not what one finds in nature has been termed “Lewontin's paradox” (Buffalo, 2021; Ellegren & Galtier, 2016) and is increasingly being considered in empirical studies (Pearse et al., 2006; Poissant et al., 2005; Randi et al., 2004; Valente et al., 2017) and theoretical models (Brandvain & Wright, 2016; Carroll et al., 2019; Evans et al., 2007; Kramer & van der Werf, 2010). The importance of population size and isolation (“insularity”) to GD thus remains uncertain and variable in nature.

One potential reason for differing results among systems—and for empirical deviations from theoretical expectations—is that insular populations can differ drastically from each other in a variety of factors influencing GD. These factors can include different evolutionary histories, effective population size, selection pressures, and demographic histories, which can lead to vastly different contemporary genetic structures (Frankham, 1995, 1996; Kuo & Janzen, 2004; Luna et al., 2007; Vega et al., 2007). Another important consideration is the extent to which populations are at or near demographic equilibrium. That is, a population sampled at a particular time might have levels of GD quite different than expected at equilibrium, potentially due to recent perturbations, such as bottlenecks or founder events (Busch et al., 2007; Schultz et al., 2009; Wereszczuk et al., 2017). Bottlenecked populations might therefore experience a slow loss of GD on the way to a new (lower) equilibrium GD (Assis et al., 2013; Ehrich & Jorde, 2005; Kuro‐o et al., 2010; Wenink et al., 1998). Under analogous conditions, among population GD might be temporarily higher (Kekkonen et al., 2011; Labonne & Hendry, 2010) or lower (Pinho et al., 2008) than expected depending on the specifics of founder effects and subsequent gene flow. These non‐equilibrium signatures can take a very long time to decay, especially in large populations (Waples, 1998; Whitlock, 1992a, 1992b). Moreover, as introduced above, organisms with “long” life histories can have an especially slow approach to a new equilibrium, with bottlenecked populations maintaining unexpectedly high GD on ecologically relevant timescales (Anijalg et al., 2020; Hailer et al., 2006; Kuo & Janzen, 2004; Stoffel et al., 2018). Even at equilibrium, different life histories (Bohonak, 1999) and movement abilities (Kisel & Barraclough, 2010) can drastically alter patterns of GD. Yet another consideration is the overall level of GD in a system. For instance, if little GD exists in an entire meta‐population, GD among populations might show a little signature of local population size or isolation (Hoelzel et al., 2002).

In summary, there are many reasons why insularity might not generate consistent effects on GD within or among populations. Therefore, it remains unclear what are the typical patterns of GD for insular populations—the very populations about which concerns regarding GD are so often raised (Moura et al., 2019; Rodríguez‐Rodríguez et al., 2019). To shed some light on this topic, we conducted a quantitative literature review based on the extent to which insular populations differ in GD from comparable non‐insular populations. The former (insular) are typically those populations found on islands, in small headwater lakes, or on mountain “sky islands,” whereas the latter (non‐insular) are often large and not isolated, such as those on the mainland, in downstream lakes, or in a large contiguous mountain range. To facilitate this analysis, we implemented a paired design, where each data point was a comparison between insular and non‐insular GD metrics for the same species within the same study. By extracting data from populations within the same studies, we were able to control for between‐study variation in methodology, as well as demographic and life history differences between focal species.

### 1.1 Hypotheses

We considered four alternative hypotheses for the effects of insularity on GD *within* populations—expressed here as they would appear on a plot of paired non‐insular (*x*‐axis) versus insular (*y*‐axis) populations.

**FIGURE 1:**
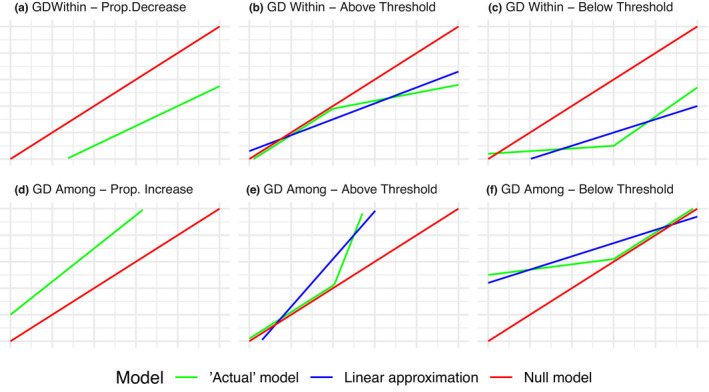
Graphical representations of predictions for each hypothesis. For each case, the red line represents the one‐to‐one line of the null model. The green line represents the model specified by the hypothesis and the blue line represents a linear approximation to the “actual” model if the model is non‐linear. In the top row, from left to right are (a) the classic model of constant, proportionally lowered genetic diversity in insular populations, (b) the alternative model of similar diversity at low values of non‐insular diversity but reduced diversity in insular populations above some critical value, (c) the alternative of greatly reduced diversity in insular populations at low values of non‐insular diversity but merely proportionally reduced diversity above some critical value. In the bottom row, (d) the classic model of average insular‐to‐non‐insular divergence always being higher than non‐insular‐to‐non‐insular divergence, (e) the alternative model of similar levels of divergence when non/non‐divergence is low but higher levels of divergence when non/non‐divergence is greater, and (f) the alternative model of highly increased divergence between insular populations and non‐insular ones when average non/non‐divergence is low but becoming approximately equal when the non/non‐divergence is already high. The above/below threshold distinction is in reference to regions where it is parallel to the one‐to‐one line

1. Insularity has no effect on GD within populations: In this case, the data (e.g., allelic richness) in a plot of paired non‐insular versus insular populations would fall on the one‐to‐one line. Meaning whatever GD levels are typical for the system as a whole, these are similar for insular and non‐insular populations (Figure 1a–c, “within—null”).
2. Insularity causes a proportional loss of GD within populations: The expectation here would be a linear relationship below the one‐to‐one line with a slope less than or equal to one (Figure 1a, “within—proportional loss”).
3. Insularity has no effect *up to* some critical value but decreases GD *after* that value: For instance, insularity might only influence GD when a certain level of GD is present overall (Figure 1b, “within—above threshold”). Under this hypothesis, the data would fall on the one‐to‐one line *up to* some threshold, but below the one‐to‐one line *after* that threshold.
4. Insularity has no effect *after* some critical value but decreases GD *up to* that value (Figure 1c, “within—below threshold”): Under this hypothesis, the data would fall on the one‐to‐one line *after* some threshold, but below the one‐to‐one line *up to* that threshold.

We considered four analogous hypotheses for the effects of insularity on GD *among* populations—expressed here as they would appear on a plot of non‐insular/non‐insular population pairs (*x*‐axis) versus insular/non‐insular population pairs (*y*‐axis) populations. Note that insular/insular population pairs were not feasible to analyze, with more details provided in Section 2.

1. Insularity has no effect on GD among populations: In this case, the data (e.g., *F*_ST_) would fall on the one‐to‐one line. Meaning that whatever the level of GD among non‐insular/insular population pairs, they are roughly the same as GD among non‐insular populations within a system (Figure 1d–f “among—null”).
2. Insularity causes a proportional increase in GD among populations: The expectation here would be a linear relationship above the one‐to‐one line with a slope greater than or equal to one (Figure 1d, “among—proportional increase”).
3. Insularity has no effect *up to* some critical value but increases GD *after* that value: That is, insularity only becomes a factor influencing GD among populations when a certain level of GD is present overall (Figure 1e, “among—above threshold”). Under this hypothesis, the data would fall on the one‐to‐one line *before* some threshold, but above the one‐to‐one line *after* that threshold.
4. Insularity has no effect *after* some critical value but increases GD *up to* that value (Figure 1f, “among—below threshold”): Under this hypothesis, the data would fall on the one‐to‐one line *after* some threshold, but above the one‐to‐one line *before* that threshold.

## 2 METHODS

### 2.1 Search methodology, inclusion criteria, and data collection

Relevant literature was identified by D.A.G.A.H. using Google Scholar between October 15, 2017 and February 23, 2018. The latter was therefore the cut‐off publication date for inclusion of any study sourced from Google Scholar. In short, various combinations of terms such as “insular” or “isolated,” along with terms such as “genetic diversity,” “genetic differentiation,” or “*F*_ST_” were used. Given the type of results produced by Google Scholar, a search was considered complete after the first 100 results had been examined and the quality of the results had declined, meaning that no papers meeting our criteria on a page of 10 results were found. This termination procedure was followed, rather than proceeding to exhaustion, given that the results of our searches usually numbered in the thousands. Although this method was not exhaustive, past literature (Mastrangelo et al., 2010) has established that Google Scholar tends to be much less specific (i.e., higher rate of inclusion of non‐relevant results) but much more sensitive (i.e., lower rate of exclusion of relevant results) than alternative search engines such as PubMed and Web of Science. Indeed, subsequent PubMed and Web of Science searches did not uncover papers that were not found by Google Scholar searches. Thus, we are confident that the papers included in this analysis are a representative sample of all available literature and the search results are repeatable based on the cut‐off date defined above.

Comparison of metrics *within* studies mitigated effects associated with searches that may not be exhaustive (above) or suffer from publication bias (Jennions & Moeller, 2002) given that we did not conduct a formal meta‐analysis (ArchMiller et al., 2015; Nakagawa et al., 2017). Formal meta‐analytic approaches also require that studies report measures of variability from which effect sizes can be calculated, but this was not the case for many studies in our database. We instead relied on conventional statistical tests (see below; also see Carlson & Seamons, 2008; Darimont et al., 2009; DiBattista, 2008; Sanderson et al., 2022). It should be noted that the primary focus of many of the included studies was not to directly interrogate the differences between insular versus non‐insular populations, and so there should be no systematic bias between these categories.

Abstracts and/or full texts of the papers were examined by D.A.G.A.H. and evaluated for inclusion according to the following criteria:

1. The study was conducted in natural populations. Domesticated or captive populations were excluded.
2. The study must have included at least three populations of a given species, with at least one population identified a priori as insular and at least two populations identified a priori as non‐insular (i.e., not separated by any known biogeographical barriers other than distance).
3. The study must have reported at least one measure of GD within populations (e.g., allelic richness or haplotype diversity) or GD among populations (e.g., *F*_ST_ or Nei's genetic distance).

Any studies where the application of these criteria was perceived by D.A.G.A.H. to be ambiguous were given a second evaluation by J.D.D.

After selection for inclusion, the following information (when available) was harvested from the papers by manual inspection: taxonomic group, year(s) of collections, habitat type (marine, terrestrial, or freshwater), number of populations sampled, location of each population sampled (name as given by the original authors as well as GPS coordinates if given), if each population was insular or not (as a binary variable), type of genetic marker sampled (microsatellite, allozyme, RFLP, or SNP), number of genetic markers analyzed, genetic diversity (mean alleles, allelic richness, heterozygosity, haplotype diversity, or nucleotide diversity) and divergence values (*F*_ST_ or related metrics such as *G*_ST_, Nei's genetic distance, Jost D, Rogers genetic distance, or estimated number of migrants), and any standard errors/deviations as applicable. Attempts were made to harvest information on census population size and effective population size (Frankham et al., 2014; Luikart et al., 2010), but too few studies reported these metrics for individual populations and so we chose not to include them here. Data were entered into a common spreadsheet and then evaluated by at least one other author for errors. All subsequent data transformation and analysis were conducted in R (R Core Team, 2018) using the packages *segmented* (Muggeo, 2008) and *MuMIn* (Bartoń, 2018). Data were visualized using *ggplot2* (Wickham, 2016).

### 2.2 Statistical transformation and analysis

We standardized the data so that each GD metric had a mean of zero and a standard deviation of one across all data of that type. This standardization then allowed different metrics (e.g., percent polymorphism and allelic richness) to be combined and compared on a common scale in subsequent analyses. Transformed data were analyzed in a paired manner, with each pair corresponding to average values of within population or among population GD within a study. For within population GD, the first value (*x*‐axis) in each pair was the value averaged for all non‐insular populations within a study, and the second value (*y*‐axis) was the comparable value averaged for all insular populations. For among population GD, the first value was the average divergence between all pairwise comparisons of non‐insular populations within a study, and the second value (*y*‐axis) was the average of all comparisons between insular populations and non‐insular populations. Note that insular to insular comparisons were not examined as they were rare; most studies considered only a single insular population.

The data were then fitted to the statistical models listed below, which were compared based on Akaike information criterion (AICc) values (Akaike, 1974) in the R package *MuMIn* (Bartoń, 2018). Included in the analysis was crude taxonomic grouping (mammals, birds, herps, fish, invertebrates, and plants), which helped improve the fit of each model. Each of these statistical models corresponded to one or more alternative patterns outlined generally in Section 1 and more specifically below in relation to our data standardization. Specific parameters of the statistical model, such as the location of the intersection in the null model with regards to the range of the data (i.e., falls inside vs. outside the range of data), are also noted below, as some alternative patterns are differentiated by these parameters. Logarithmic (for within population GD) and exponential (for among population GD) transformed models were used as curved approximations to broken stick models as a contingency if broken stick models were unable to be fit. Given that some models involved logarithmic transformation, all data were increased by a constant so that the minimum value across the entire data set was 1 (as opposed to a negative number). Both axes were increased by the same constant so that ultimate intercepts and shapes would be maintained.

### 2.3 Models for GD within populations

1. Null model: insular value ~ taxon group + non‐insular value (fixed slope of 1 and fixed intercept of 0, i.e., on the one‐to‐one line). This statistical model is consistent with the “within—null” hypothesis. See Section 1 for details of all hypotheses.
2. Semi‐null model: insular value ~ taxon group + non‐insular value (fixed intercept of 0, free slope, i.e., same intercept as the one‐to‐one line). This statistical model is consistent with the “within—proportional loss” hypothesis.
3. Linear model: insular value ~ taxon group + non‐insular value (free slope and intercept). This statistical model is consistent with the “within—proportional loss,” “within—above threshold,” or “within—below threshold” hypotheses, depending on parameter values.
4. Log transformed model: insular value ~ taxon group + log(non‐insular value). This statistical model is consistent with the “within—above threshold” hypothesis.
5. Exponential transformed model: insular value ~ taxon group + exp(non‐insular value). This statistical model is consistent with the “within—above threshold” hypothesis.
6. “Broken stick” model: linear model as per number three but segmented into two regions of non‐insular value utilizing the R package *segmented* (Muggeo, 2008). This statistical model is consistent with the “within—above threshold” or “within—below threshold” hypotheses, depending on the breakpoint and direction of segments.

### 2.4 Models for GD among populations

1. Null model: (insular to non‐insular comparison) ~ (non‐insular to non‐insular comparison) (fixed slope of 1 and fixed intercept of 0, i.e., on the one‐to‐one line). This statistical model is consistent with the “among—null” hypothesis.
2. Semi‐null model: (insular to non‐insular comparison) ~ (non‐insular to non‐insular comparison) (fixed intercept of 0, free slope, i.e., same intercept as the one‐to‐one line). This statistical model is consistent with the “among—proportional increase” hypothesis.
3. Linear model: (insular to non‐insular comparison) ~ (non‐insular to non‐insular comparison) (free slope and intercept). This statistical model is consistent with the “among—proportional increase,” “among—above threshold,” or “among—below threshold” hypotheses, depending on parameter values.
4. Exponential transformed model: (insular to non‐insular comparison) ~ exp(non‐insular to non‐insular comparison). This statistical model is consistent with the “among—proportional increase” hypothesis.
5. “Broken stick” model: linear model as per #3, but segmented into two regions of non/non‐value utilizing the R package *segmented* (Muggeo, 2008). This statistical model is consistent with the “among—above threshold” or “among—below threshold” hypotheses, depending on the breakpoint and direction of segments.

## 3 RESULTS

**FIGURE 2:**
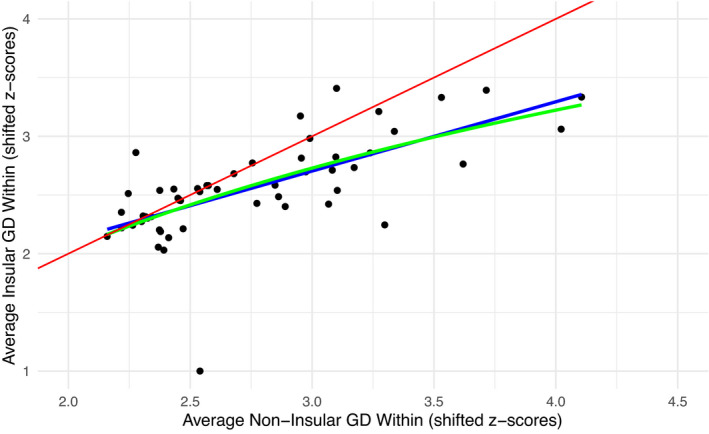
Genetic diversity (GD) within populations. Each point represents an average of all GD metrics reported in a study for all non‐insular populations (*x*‐axis) and insular populations (*y*‐axis). The red line represents a null model of a one‐to‐one relationship. The colored lines represent the trendlines for the two fitted models, both of which were preferred >10,000 times to the null model by AICc comparison. The green line represents the log‐transformed model (relative weight 0.571) and the blue line represents a linear model (relative weight 0.429). This results in the log‐transformed model being preferred to the linear model by only 1.33 times. Note that the intercept of the null and linear models occurs within the range of data (intercept ≈2.31, range 2.16–4.71)

For within population GD, the “within—above threshold” hypothesis (Figure 1b) was favored by the data (Figure 2). That is, the data indicate that insularity has no effect on GD below a certain level, but above that level, insularity results in decreased within population GD relative to non‐insular counterparts. In support of this conclusion, the AICc comparisons selected the log‐transformed and linear models (see “Section 2.3”) equally, both of which are consistent with that hypothesis. Also, the specifics of the linear model, namely the location of the intercept between the linear and null models relative to the range of data (intercept ≈2.31, range 2.16–4.71), were in line with the predictions of the within—above threshold effect. Figure 1b shows how the intercept falling within this range is indicative of this hypothesis as opposed to the “proportional loss” hypothesis in Figure 1a. All alternate models presented were excluded by model selection or model parameters (Table 1). We note that the broken stick model—which is the most intuitive model for a threshold hypothesis—did not fit the data. Indeed, the *segmented* package estimated the breakpoints at the boundary of the data, indicating that segmenting the data set did not improve fit under any estimate of a breakpoint (i.e., a straight linear fit was always better). Thus, we instead infer the threshold hypothesis from the linear and log‐transformed models, which approximate the broken stick model without the necessity of estimating a breakpoint.

**TABLE 1:** Model comparison for the within population genetic diversity analysis. Model weights were calculated by the R package *MuMIn*. Details of hypotheses are presented in Section 2

Model description	Corresponding hypothesis	ΔAICc value	Relative model weight
Log‐transformed	Within—above threshold effect	—	0.571
Linear	Within—proportional gain, Within—above threshold effect, within—below threshold effect (depending on specific parameters)	0.6	0.429
Exponential‐transformed	Within—below threshold effect	13.1	0.001
Semi‐null	Within—below threshold effect	25.4	<0.001
Null	Within—null	25.4	<0.001

**FIGURE 3:**
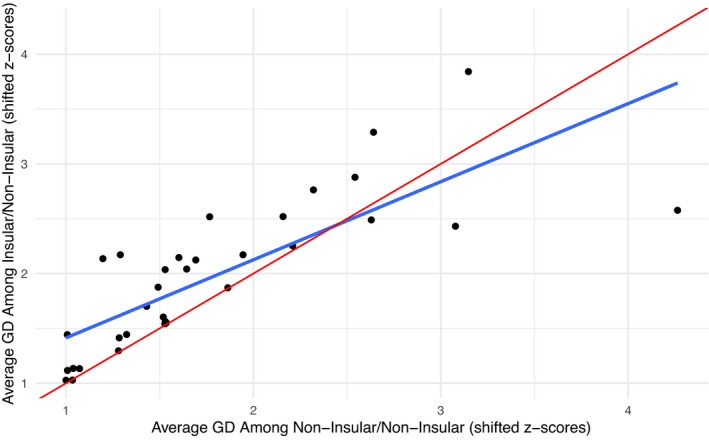
Genetic diversity (GD) among populations. Each point represents an average of all GD metrics within a study for all non‐insular/non‐insular comparisons (*x*‐axis) and all insular/non‐insular comparisons (*y*‐axis). The red line represents a null model of a one‐to‐one relationship. The blue line represents the trendline for the fitted linear model, which was preferred 84.7 times to the null model by AICc comparison (0.932 vs. 0.011 relative model weights). Note that the intercept of the null and linear models occurs within the range of data (intercept ≈2.44, range 1.00–4.26), and the slope of the linear model is less than 1 (slope = 0.7014)

For among population GD, the data (Figure 3) generally supported the “among—below threshold” model, where insularity has no detectable effect on among population GD above a certain level of GD, but below a certain level, insularity results in increased among population GD relative to non‐insularity. However, the evidence was much less conclusive than that observed for within population GD as described above. In the above GD‐within analysis, two models were roughly equal, one of which was consistent only with the preferred hypothesis, and the other was consistent with multiple hypotheses, but parameter values made it consistent with the preferred hypothesis only. Here for the GD‐between analysis, only one model was preferred, and it was consistent with multiple hypotheses depending on parameter values (Table 2). The broken stick model—again the most intuitive model—did not fit, since the *segmented* package estimated the breakpoints at the boundary of the data, interpretable as that segmenting the data set did not improve fit under any estimate of a breakpoint (i.e., a straight linear fit was always better).

**TABLE 2:** Model comparison for among population genetic diversity analysis. Model weights were calculated by the R package *MuMIn*. Details of hypotheses are presented in Section 2

Model description	Corresponding hypothesis	ΔAICc value	Relative model weight
Linear	Among—proportional increase, among—above threshold, among—below threshold (depending on specific parameters)	—	0.932
Semi‐null	Among—proportional increase	5.6	0.057
Null	Among—null	8.9	0.011
Exponential‐transformed	Among—above threshold, among—below threshold (depending on specific parameters)	23.8	<0.001

## 4 DISCUSSION

Based on the existing literature, we would expect insular populations to typically have lower GD than otherwise equivalent non‐insular populations of conspecifics. In our study, we did not observe this trend based on our data extracted from the literature (Figures 2 and 3). Instead, we found that insularity appears to reduce within population GD only when GD levels are already generally high in the system. Similarly, we found that insularity increases among population GD only when GD levels are otherwise low, and even then, the difference is small and only weakly supported. These observations therefore lead to several important considerations.

First, although insularity can reduce GD within populations and increase GD among them, other factors can—and often do—play a role in the outcome. Figure 2, for instance, demonstrates that within population GD varies dramatically among study systems; yet this variation is mostly along the one‐to‐one line, which suggests that insularity often has comparatively little effect on GD compared to other factors. Indeed, 23 of the 54 studies in our analysis yielded average insular within population GD that was within the 95% confidence interval of the one‐to‐one line, another five studies had insular within population GD *above* that confidence interval, leaving only 26 of 54 studies have significantly reduced average within population GD compared to the null expectation. Of course, these latter cases of reduced GD in insular systems are often striking, suggesting that theoretical expectations do often apply. For instance, Iguchi and Nishida (2000) found greatly reduced within population GD in an insular amphidromous fish (Ayu or *Plecoglossus altivelis*) based on mitochondrial DNA. These patterns for within population GD are largely mirrored by among population GD, although the effects are weaker for the latter (Figure 3). As an example, Álvarez‐Castañeda and Murphy (2014) found that even though most islands populations of a rodent (Spiny Pocket Mouse or *Chaetodipus spinatus*) were highly divergent from those on the nearby mainland peninsula, at least one population was not statistically different. Thus, while theoretical expectations do often apply, notable counterexamples can even exist within a single study. As we noted in Section 1, other recent investigators (Ellegren & Galtier, 2016) also concluded that insularity is not an overwhelming driver of low GD: “Life history, but not population history, predicts genetic diversity,” although this statement firmly places GD estimates in the context of species instead of individuals or populations.

Second, the instances where insularity did in fact influence GD were not randomly distributed. On the contrary, insularity had its greatest effects when GD was relatively high in a system's non‐isolated populations. Although theoretical work (Charlesworth, 2009) suggests that systems with low GD should experience the strongest effects of insularity, our analysis suggests these systems are the most constrained. Specifically, in a system with low GD overall, little scope exists for GD to further decrease. Instead, a greater scope seems to exist for the effects of insularity on GD when GD is higher overall within a given study system (Figure 2). As an example, Zhao et al. (2014) found that the Eastern Honeybee (*Apis cerana*), with low overall GD, had island populations that were not any less diverse than mainland ones. In contrast, Álvarez‐Castañeda and Murphy (2014) found a great deal of variation in GD within populations, but this trend was embedded in a system already rich with haplotypes.

A potential explanation for why our analysis did not fully support theoretical expectations for the effects of insularity is that even non‐insular populations might have recently undergone reductions in population size and gene flow, making them—in essence—also insular. However, the studies in our dataset did not generally report this kind of population decline or fragmentation in their non‐insular populations. Furthermore, declines in GD following bottlenecks can be slow with increasing generation times (Anijalg et al., 2020; Hailer et al., 2006; Kuo & Janzen, 2004; Stoffel et al., 2018), and so we would not expect populations experiencing recent bottlenecks to show noteworthy changes in GD. Indeed, several studies have shown only small decreases in GD with recent and sometimes severe population declines (Leigh et al., 2019; Millette et al., 2020). Therefore, we come back to our conclusion that insularity has its greatest effect on GD when GD is high within a system since that is when there is the most variability for the effects of insularity to manifest themselves. Finally, the effects of insularity appear to be more apparent for within population GD (Figure 2) versus among population GD (Figure 3). This finding might simply reflect sample size: only 34 studies passed all criteria for inclusion in the among population analyses, as opposed to 54 studies for the within population analyses.

### 4.1 Overall implications

Our analysis suggests that classical assumptions about the genetic consequences of small population size and isolation (together “insularity”) are not universal in natural populations, although we could not explicitly model the effects of population size due to insufficient information in most published studies. This ambiguity supports discussions expressed in recent reviews (Ellegren & Galtier, 2016; Meirmans & Hedrick, 2011). Thus, empirical assessment of GD should be a requirement to conclude that insular pockets of a larger meta‐population are genetically distinct and characterized by low GD. Of course, this statement does not mean that such populations are unworthy of the special assessment or concern as these populations might be particularly sensitive to the risks of insularity depending on their life histories (Coleman et al., 2018). We therefore agree with the statement that “island populations should have less genetic variation than mainland populations” (Frankham, 1996), but also caution that this needs to be explicitly tested in each case.

Genetic diversity is increasingly highlighted as a level of biological diversity well worth targeted conservation efforts (Des Roches et al., 2018; Mimura et al., 2017). Indeed, GD can enable adaptive evolutionary responses to rapidly changing environments or even “rescue” populations suffering from fitness declines (Carlson et al., 2014; Hendry et al., 2018; Whiteley et al., 2015). Based on our findings, we caution against inferring low GD for insular populations, as this may not always be the case (Leigh et al., 2019; Millet et al., 2019). Populations are also often defined as insular based on their geographic isolation, small effective population size, or reductions in gene flow, but again, these assumptions and their direct effects on GD may not have been explicitly tested. Moreover, the convergence of GD on any new equilibrium can be quite slow (Anijalg et al., 2020; Hailer et al., 2006; Kuo & Janzen, 2004; Stoffel et al., 2018), meaning that populations that meet all of the criteria above may not respond as expected on ecological time scales. Given only a few common conditions, such as “long” life histories, severe disturbances that will ultimately result in declines in GD might have no obvious immediate effect on some groups of animals, but severely impact others. For example, adult lifespan appeared to be the best predictor of GD based on the whole genome analysis of 16 European marine fish species, but downstream simulations demonstrated that this relationship did not hold for birds and mammals (Barry et al., 2022). This lag seems analogous to the concept of “extinction debt” (Tilman et al., 1994), where changing environmental conditions will ultimately result in the extinction of species, and yet they persist on sampling timescales, even if they are in a terminal decline. Here, a population may have “homogeneity debt,” where conditions will ultimately reduce their GD, yet there has not been sufficient time to make this apparent. In short, dramatic, even catastrophic decreases in GD might be inevitable, even when these effects are not apparent in the data.

## CONFLICT OF INTEREST

The authors declare no competing interests.

## AUTHOR CONTRIBUTIONS

**David A. G. A. Hunt:** Conceptualization (lead); Data curation (lead); Formal analysis (lead); Investigation (lead); Methodology (lead); Project administration (lead); Validation (lead); Visualization (lead); Writing – original draft (lead); Writing – review & editing (supporting). **Joseph D. DiBattista:** Conceptualization (supporting); Data curation (supporting); Formal analysis (supporting); Investigation (supporting); Methodology (supporting); Project administration (supporting); Supervision (supporting); Validation (supporting); Visualization (supporting); Writing – original draft (supporting); Writing – review & editing (lead). **Andrew P. Hendry:** Conceptualization (supporting); Data curation (supporting); Formal analysis (supporting); Funding acquisition (lead); Investigation (supporting); Methodology (supporting); Project administration (supporting); Supervision (supporting); Validation (supporting); Visualization (supporting); Writing – original draft (supporting); Writing – review & editing (supporting).

## Data Availability

Raw data and code (R script) to reproduce the results are available at Dryad: https://doi.org/10.5061/dryad.kwh70rz62.
